# Expression of AIB1 protein as a prognostic factor in breast cancer

**DOI:** 10.1186/1477-7819-9-139

**Published:** 2011-10-29

**Authors:** Kyungji Lee, Ahwon Lee, Byung Joo Song, Chang Suk Kang

**Affiliations:** 1 Department of Hospital Pathology, College of Medicine, The Catholic University of Korea, 505 Banpo-dong, Seocho-Gu, Seoul 137-701, Korea; 2 Department of Surgery, College of Medicine, The Catholic University of Korea, 505 Banpo-dong, Seocho-Gu, Seoul 137-701, Korea

**Keywords:** breast, neoplasm, AIB1, immunohistochemistry, *in situ *hybridization

## Abstract

**Background:**

AIB1 (amplified in breast cancer I) is a member of the p160 steroid receptor coactivator family. AIB1 is frequently overexpressed in breast cancer and has functions that promote oncogenesis that are independent of estrogen receptor (ER) coactivation. We investigated prognostic significance of AIB1 and relationship between AIB1 and ER, progesterone receptor (PR), androgen receptor (AR), DAX-1, and HER2.

**Methods:**

RNA *in situ *hybridization (ISH) and immunohistochemical (IHC) staining for AIB1, IHC staining for ER and the progesterone receptor (PR) and IHC staining and silver in situ hybridization (SISH) for HER2 were performed for 185 breast cancer cases.

**Results:**

A high level of expression of AIB1 mRNA was observed in 60.0% of tumors. IHC analysis detected AIB1 positivity in 47.3% of tumors, which did not correlate with AIB1 mRNA expression (p = 0.24, r = 0.10). AIB1 protein expression correlated with AR and DAX-1 expression (p = 0.01, r = 0.22 and p = 0.02, r = 0.21, respectively) but not with ER or PR expression (p = 0.14, r = -0.13 and p = 0.16, r = -0.12, respectively). AIB1 protein expression correlated with the amplification of the HER2 gene (p = 0.03, r = 0.19). In contrast to AIB1 protein expression, AIB1 mRNA expression did not correlate with AR, DAX-1, ER, and PR expression, and the amplification of the HER2 gene (p > 0.05 for all).

There were trends that strong AIB1 protein expression correlated with poorer disease free survival (p = 0.07). Strong AIB1 protein expression correlated with poorer overall survival (p = 0.04). Among the ER-negative subgroup, strong AIB1 protein expression correlated with poorer disease free survival and overall survival (p = 0.01 and p < 0.01, respectively).

**Conclusions:**

Strong AIB1 protein expression was poor prognostic factor in breast cancer, especially in ER-negative breast cancers. Further investigation is essential to determine whether AIB1 might be effective therapeutic targets for ER-negative breast cancers.

## Background

Breast cancer is the most frequently diagnosed cancer and the leading cause of cancer death among females, accounting for 23% of all cancers [1]. Estrogen hormones regulate the development and growth of normal and malignant breast epithelial cells via the estrogen receptor (ER). Selective estrogen receptor modulators such as tamoxifen are well-established treatment modalities for ER-positive breast cancer. However, a significant proportion of ER-positive patients suffer from endocrine therapy resistance. Furthermore, up to 30% of breast cancers are negative for ER, lacking effective targeted therapy [2].

Estrogen signaling are the interactions of ER with transcriptional coactivators including p/CAF, CREB binding protein(CBP), p300 and the p160 family members [3]. AIB1 (amplified in breast cancer I) is a member of the p160 steroid receptor coactivator family, which includes SRC-1 and SRC-2 [4]. It is located on chromosome 20q12, a common region of amplication in breast cancer. It is recruited to hormone-responsive genes through their interaction with activated receptors and then nucleate the assembly of a coactivator complexs, which in turn remodels chromatin through histone modifications and facilitates RNA polymerases II transcription [5]. AIB1 can activate nuclear receptors such as ER and the androgen receptor (AR) and transcription factors such as E2F1 [6]. Anzick et al. have demonstrated that the AIB1 gene is overexpressed in breast cancer and ovarian malignancy [5]. Other previous studies have indicated that AIB1 has important roles in carcinogenesis in breast tissue and is associated with resistance to endocrine treatment [5,7]. AR, which is detected in ER-negative breast cancer, has recently been suggested as a therapeutic target for a subset of triple-negative breast cancers [8]. AIB1 is a preferred co-activator for AR in prostate cancer [9].

In this study, we investigated the prognostic significance of AIB1 and its relationship with steroid hormone receptors including ER, the progesterone receptor (PR), and AR, DAX-1, and HER2.

## Materials and methods

### Patients and tissue samples

We analyzed 185 patients who underwent surgical resection and had confirmed breast carcinoma between January 2004 and December 2008 at the Kangnam St. Mary's Hospital at the Catholic University of Korea. The normal tissue of breast, palatine tonsil, placenta and pancreas for control were obtained after anonymization. This study was approved by the hospital's Institutional Review Board and followed recommendations for tumor marker prognostic studies [10]. The mean age at diagnosis was 51 years (range, 30-71 years). Most tumors (n = 173) were invasive ductal carcinomas. The remaining tumors were 3 cases of invasive lobular carcinoma, 4 cases of mucinous carcinoma, 2 cases of medullary carcinoma, 1 tubular carcinoma and 2 invasive micropapillary carcinoma. Data regarding patient demographics were obtained by reviewing medical records. The immunohistochemical (IHC) staining data for ER, PR, AR, DAX-1, and HER2 were obtained from a previous study in 133 overlapping patients [11]. The median length of follow-up was 52.5 months (range, 4.2-89.8 months). Within the observation period, there were 5 breast cancer-specific deaths and 22 breast cancer relapses. The other clinicopathologic characteristics are summarized in Table 1.

**Table 1:** Correlation of clinicopathological parameters and AIB1 mRNA expression in 185 breast cancer patients

		AIB1 mRNA (n = 185)		
			
		low	high	p-value
Age				
	≤ 50	59	39	
	> 50	52	35	0.54

Histological type				
	IDC	107	66	
	non-IDC	4	8	0.05

Histological grade				
	well	41	15	
	moderate	47	41	
	severe	23	18	0.05

T stage				
	T1	69	29	
	T2	38	41	
	T3	4	4	**0.01**

Lymph node metastasis				
	negative	59	46	
	positive	51	28	0.16

Stage				
	T1	48	23	
	T2	41	37	
	T3	19	14	
	T4	3	0	0.13

ER				
	negative	30	27	
	positive	81	47	0.12

PR				
	negative	31	27	
	positive	80	47	0.14

HER2				
	negative	85	53	
	positive	26	21	2.28

AR				
	negative	19	5	
	positive	67	38	0.11

DAX-1				
	negative	25	7	
	positive	61	36	0.08

AIB1: amplified in breast cancer I; IHC: immunohistochemistry; IDC: invasive ductal carcinoma; ER: estrogen receptor; PR: progesterone receptor; and AR: androgen receptor.

### Tissue microarray

To construct the tissue microarray block, 2 mm-sized core biopsies were taken from morphologically representative areas of formalin-fixed and paraffin-embedded tumor tissue and were assembled on a recipient paraffin block containing 30 biopsies using a precision instrument (Micro Digital Co., Gunpo-si, Gyeonggi-do, Korea). After construction, 4 μm sections were removed, and the histology was verified by hematoxylin-eosin staining. Each of the recipient blocks included one core of normal breast for internal control, two cores of palatine tonsil and placenta for proper TMA orientation and universal control.

### mRNA *in situ *hybridization (ISH)

**Figure 1:**
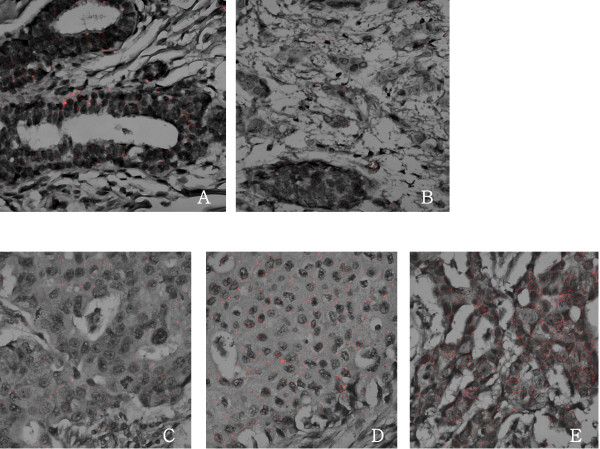
**RNA in situ hybridization for AIB1 mRNA**. A: Expression of AIB1 mRNA was 1+ in normal breast tissue samples. B: Breast cancer tissue sample with 1+ AIB1 mRNA expression (×400). B: Breast cancer tissue sample with negative AIB1 mRNA expression (×400). C: Breast cancer tissue sample with low (1+) AIB1 mRNA expression (×400). D: Breast cancer tissue sample with high (2+) AIB1 mRNA expression (×400). E: Breast cancer tissue sample with high (3+) AIB1 mRNA expression (×400).

A commercially available mRNA ISH kit (QuantiGene^®^ViewRNA, Paranomics Inc., Fremont, CA, USA) was used according to the manufacturer's protocol. Five micrometer sections for the paraffin embedded tissue arrays were cut and attached to positively charged glass slides. The samples were incubated with a pretreatment solution followed by protease digestion. An AIB1-gene-specific probe was designed (forward primer: CTAATCCCTATGGCCAAGCA and reverse primer: CTTTCGTCACTCTGGCCTTC). A probe set was hybridized and amplifier molecules were hybridized to each pair of oligonucleotides. The fast red substrate, alkaline phophatase breaks down the substrate to form a precipitate. AIB1 mRNA is visualized using confocal microscopy (LSM 510 Meta, Carl Zeiss, Oberkochen, Germany). AIB1 mRNA was independently examined by two pathologists. The expression level was semi-quantitatively determined based on the number of cytoplasmic red dots in the tumor cells (0, 1+, 2+, and 3+). In normal breast tissue, the expression of AIB1 mRNA was 1+. For the purpose of further analysis, the data were organized into two categories using a cut-off value equal to the AIB1 mRNA level in normal breast: low expression (0 and 1+) and high expression (2+ and 3+) (Figure 1).

### Immunohistochemistry (IHC)

Four-micrometer sections of the formalin-fixed and paraffin-embedded tissue arrays were deparaffinised and rehydrated in a graded series of alcohol. Endogenous peroxidase activity was inhibited using 3% hydrogen peroxide. Heat-induced epitope retrieval was conducted by immersing the slides in Coplin jars that were filled with 0.01 M citrate buffer (pH6.0), boiling the slides in a microwave vacuum histoprocessor (RHS-1, Milestone, Bergamo, Italy) at a controlled final temperature of 121°C for 15 min and cooling the slides to room temperature for 15 min. The tissue arrays were processed in an automatic IHC staining machine (Lab Vision Autostainer, Lab Vision Co., Fremont, CA, USA) with a DAKO ChemMate™ EnVision™ system (DAKO, Carpinteria, CA, USA) according to the manufacturer's protocol. The following antibodies were used: AIB1 (1:100, NCoA-3, Santa cruz biotechnology), ER (1:100, 6F11, Novocastra, Newcastle, UK), PR (1:50, PgR636, DAKO), and HER2 (1:200, polyclonal, DAKO). The immunoreactions were visualized with 3-3'-diaminobenzidine (DAB) and counterstained with Mayer's hematoxylin. Positive control for AIB1 was paraffin embedded pancreas tissue, which showing cytoplasmic staining of exocrine glands [12].

**Figure 2:**
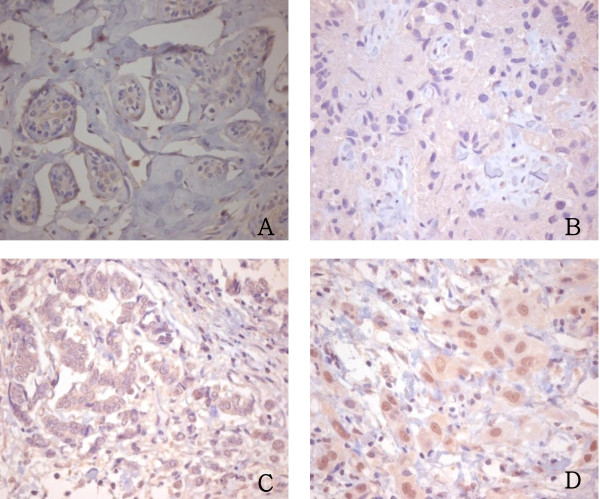
**Immunohistochemical staining for AIB1**. A: Expression of AIB1 was negative in normal breast tissue samples (×200). B: Breast cancer tissue sample with negative AIB1 staining (×200). C: Breast cancer tissue sample with weak AIB1 staining (×200). D: Breast cancer tissue sample with strong AIB1 staining (×200).

Immunostaining for AIB1, ER, PR and HER2 was independently examined by two pathologists. The tumors with > 10% nuclear-stained cells were considered positive for AIB1, ER, or PR. For AIB1, their staining intensities were classified into three categories: 0, negative; 1+, weakly positive, and 2+, strong positive (Figure 2). For ER and PR (including previously stained overlapping cases [11]), the proportion of positively stained cells was classified into 5 categories: 0, negative; 1+, 11-25%; 2+, 26-50%; 3+, 51-75%; 4+, 76-100%. The HER2 expression level was classified into four groups according to the ASCO/CAP guideline recommendations for HER2 IHC [13].

### HER2 silver in situ hybridization (SISH)

The HER2 status was confirmed using HER2 SISH, when the IHC staining result for HER2 was equivocal (2+). Four-micrometer sections of the tissue arrays were stained according to the manufacturer's protocols with the INFORM HER2 DNA probes (Ventana, Tucson, USA). The probe was labeled with dinitrophenol (DNP) and optimally formulated for use with the ultraView SISH Detection Kit and the Ventana BenchMark^®^XT automated slide stainer (Ventana). The black dot signals for the HER2 gene were counted in at least 20 tumor cells and classified into 3 categories: negative if HER2 signals/nucleus < 4, equivocal if HER2 signals/nucleus 4-6, and positive if HER2 signals/nucleus > 6.

### Statistical analysis

All statistical analyses were performed using SPSS (version 13.0, SPSS Inc., Chicago, IL, USA) for Windows. The association between mRNA ISH and IHC results and clinicopathological variables was evaluated using the Chi-square test or Fisher's exact probability test. The association between AIB1 and steroid hormones and co-factors was evaluated using the Spearman correlation test. Kaplan-Meier plots were used to estimated recurrence free survival and overall survival, and the statistical significance was determined by the log-rank test. All Kaplan-Meier curves were curtailed when less than five individuals remained at risk. *P *values less than 0.05 were considered significant.

## Results

### AIB1 mRNA expression does not correlate with AIB1 protein expression

A total of 6 (3.2%), 105 (56.8%), 50 (27.0%) and 24 (13.0%) tumors had 0, 1+, 2+, and 3+ of AIB1 mRNA, respectively (Figure 1). Using a cut-off value equal to the AIB1 mRNA in normal breast tissues (1+), we determined that 60.0% of tumors had low levels (0 and 1+) of AIB1 mRNA expression and 40.0% of tumors had high levels (2+ and 3+). Palatine tonsil and placenta showed high expression (3+) and low expression (0) of AIB1 mRNA, respectively. Using IHC analysis, we detected AIB1 positivity in 47.3% (61 out of 129) of tumors, including 46 weakly positive and 15 strongly positive cases (Figure 2). Normal breast was not stained, and palatine tonsil and placenta were weakly positive and negative, respectively. AIB1 mRNA expression did not correlate with the IHC result for AIB1 (p = 0.24, r = 0.10). High expression of AIB1 mRNA correlated with a larger tumor size (p = 0.01) (Table 1).

### AIB1 protein expression correlates with AR, DAX-1, and HER2 expression

Using a cut-off value as previously described [11], the positive rates for ER, PR, AR, and DAX-1 were 69.2%, 68.6%, 81.4% and 75.2%, respectively. AIB1 protein expression correlated with histologic type (p = 0.04) (Table 2). HER2 was amplified in 25.4% of tumors (47 out of 185). AIB1 protein expression correlated with AR and DAX-1 expression (p = 0.01, r = 0.22 and p = 0.02, r = 0.21, respectively) but not with ER or PR expression (p = 0.14, r = -0.13 and p = 0.16, r = -0.12, respectively) (Table 3). AIB1 protein expression correlated with the amplification of the HER2 gene (p = 0.03, r = 0.19) (Table 3). In contrast to AIB1 protein expression, AIB1 mRNA expression did not correlated with AR, DAX-1, ER, and PR expression, and the amplification of the HER2 gene (p > 0.05, each).

**Table 2:** Correlation of clinicopathological parameters and AIB1 protein expression in 129 breast cancer patients

		AIB1 protein (n = 129)		
			
		negative	positive	p-value
Age				
	≤ 50	36	34	
	> 50	32	27	0.44

Histological type				
	IDC	63	61	
	non-IDC	5	0	**0.04**

Histological grade				
	well	23	17	
	moderate	31	31	
	severe	14	13	0.76

T stage				
	T1	40	38	
	T2	26	22	
	T3	2	1	0.84

Lymph node metastasis				
	negative	41	33	
	positive	27	27	0.34

Stage				
	T1	33	24	
	T2	17	26	
	T3	17	9	
	T4	1	2	0.13

ER				
	negative	18	21	
	positive	50	40	0.22

PR				
	negative	18	23	
	positive	50	38	0.12

HER2				
	negative	55	39	
	positive	13	22	**0.02**

AR				
	negative	17	7	
	positive	51	54	**0.03**

DAX-1				
	negative	23	9	
	positive	45	52	**0.01**

AIB1: amplified in breast cancer I; IHC: immunohistochemistry; IDC: invasive ductal carcinoma; ER: estrogen receptor; PR: progesterone receptor; and AR: androgen receptor.

**Table 3:** Association analyses of IHC results of AIB1, ER, PR, AR, and DAX-1, and the HER2 gene status

	AIB1	ER	PR	AR
ER	-0.13/0.14	-	0.51/< 0.01	0.38/< 0.01
PR	-0.12/0.16	0.51/< 0.01	-	0.31/< 0.01
AR	0.22/0.01	0.38/< 0.01	0.31/< 0.01	-
DAX-1	0.21/0.02	0.31/< 0.01	0.26/< 0.01	0.50/< 0.01
HER2	0.19/0.03	-0.33/< 0.01	-0.34/< 0.01	-0.16/0.08

The ratios represent r-value/p-value. AIB1: amplified in breast cancer I; ER: estrogen receptor; PR: progesterone receptor; and AR: androgen receptor.

### Strong AIB1 protein expression is a poor prognostic factor in breast cancers

**Figure 3:**
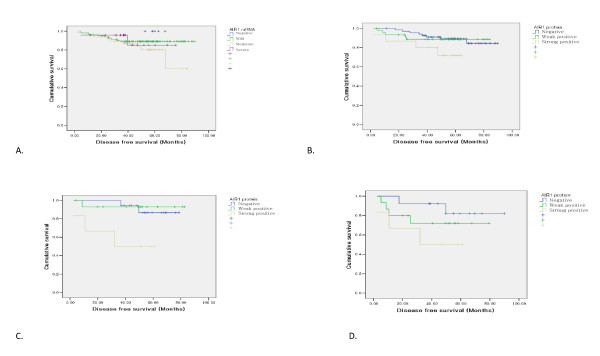
**Kaplan-Meier survival curves to evaluate differences in disease-free survival**. A: DFS according to the AIB1 mRNA expression level. B: DFS according to the AIB1 protein expression level. C: DFS according to the AIB1 protein expression level in ER-negative breast cancers. D: DFS according to AIB1 protein expression in HER2 positive breast cancers.

Disease-free survival lengths and overall survival lengths according to the AIB1 mRNA levels were not significantly different (p = 0.35 and p = 0.49, respectively) (Figure 3A). There were trends that strong AIB1 protein expression correlated with poorer disease free survival (p = 0.07) (Figure 3B). Strong AIB1 protein expression correlated with poorer overall survival (p = 0.04). In the ER-negative subgroup, strong AIB1 protein expression correlated with poorer disease free survival and overall survival (p = 0.01 and p = < 0.01, respectively) (Figure 3C). For the HER2-amplified subgroup, strong AIB1 protein expression did not have a significant impact on patient survival time (P > 0.05), although mean survival time was reduced (Figure 3D).

## Discussion

AIB1 is a well-known transcriptional coactivator that promotes the transcriptional activity of multiple nuclear receptors such as ER, PR, the thyroid hormone receptor and the retinoic acid receptor [5,14,15] AIB1 has also been shown to coactivate other hormone receptor-independent transcription factors such as STAT and AP-1 via histone acetylation/methylation [16]. Previous reports have indicated that AIB1 regulates other signaling pathways such as the PI3K/Akt/mTOR pathway [17]. AIB1 transgenic mice (overexpressing AIB1 in mammary glands) with ovariectomy, display a high incidence of mammary tumors (48%, 48/100), and AIB1 has functions that promote oncogenesis that are independent of ER coactivation [17]. Therefore, further investigation is required to ascertain the oncogenic role of AIB1 in breast cancer independent of ER coactivation.

AIB1 gene (20q13) amplication occurred in 2-10% of breast cancer smaples with high AIB1 mRNA expression. However, increased AIB1 mRNA levels have been reported in 31-64% of breast cancer [18,19], suggesting that another mechanism mediates AIB1 overexpression independent of amplification. In our study, 40.0% of tumors exhibited high expression of AIB1 mRNA and 47.3% of tumors exhibited positive AIB1 staining by IHC analysis. Previous studies have reported that AIB1 protein and mRNA levels correlate well [7]. However, our results do not confirm those reports. We showed that the AIB1 mRNA level did not correlated with AIB1 protein level, which was detected using IHC analysis (p = 0.24, r = 0.10). AIB1 protein levels are affected by various factors including the levels of AIB1 mRNA expression and stability; the translational activity, which may be regulated by exogenous substances such as tamoxifen and endogenous microRNAs such as mir-17-5p; and proteasomal degradation [7,20-22].

AIB1 is an important oncogene in breast tissue and is associated with poorer disease-free survival [23,24]. AIB1, like the ER itself, is phosphorylated and thereby fuctionally activated by MAPKs. Therefore high levels of activated AIB1 could reduce the antagonist effects of tamoxifen [3]. Clinical studies by Osborne et al. [25] and Alkner et al. [26] also reported that high AIB1 was associated with tamoxifen resistance. In the same context, it could be partly explained by resistance to antiestrogen therapy that strong AIB1 protein expression was correlated with poorer overall survival. However, there seems to be another mechanism involved other than resistance to antiestrogen therapy, because in the ER-negative subgroup which did not get antiestrogen therpy, strong AIB1 protein expression also correlated with short disease-free and overall survival (p = 0.006 and p = 0.001, respectively). Harigopal et al. have reported that AIB1 in ER-negative breast cancer is associated with a negative prognostic effect [24]. In prostate cancer, Zou et al. demonstrated the effect of AIB1 and AR depletion by adenovirus vector-mediated siRNA expression on cell proliferation [27]. Reduction of AIB1 and AR level results in inhibition of androgen dependent and androgen-independent tumor cell proliferation through direct control of cell cycle genes, suggesting that AIB1 and AR may play important roles in androgen ablation resistance by controlling cell cycle gene expression [27]. Although many breast cancers express AR, the association of AR and AIB1 in breast cancer has not been studied well. AIB1 binds specifically to AR via the first and third LXXLL motifs in AIB1 and to ER via the second LXXLL motif in AIB1 [9]. Interestingly, phosphorylation is required for AIB1 activity. AIB1 is selectively phosphorylated when cells are treated with androgen and estrogen [28].

DAX-1 functions as a global negative regulator of steroid hormone production. However, the expression of DAX-1 is positively correlated with the expression of AR in breast cancer [29]. We previously reported that DAX-1 is positively correlated with AR. In our previous report, we suggested that DAX-1 might be a more effective target than AR in triple-negative breast cancer because the overall expression rate of DAX-1 is high, even in these triple-negative breast cancers [11]. In the present study, AIB1 protein expression correlated with the expression of the nuclear receptor AR and DAX-1. Furthermore, positive IHC staining for AIB1 was observed in 53.8% of ER-negative breast cancers. These findings support the hypothesis that AR, DAX-1, and AIB1 might be effective therapeutic targets, especially for ER-negative cancers.

In the HER2-amplified subgroup, patients with strong AIB1 protein expression showed reduced disease-free survival according to the Kaplan-Meier plot (not statistically significant). Spears et al. have demonstrated tumors that overexpress both HER2 and AIB1 have poorer prognosis than HER2 and AIB1 only overexpressing tumors [30].

## Conclusions

Strong AIB1 protein expression was correlated with poorer overall survival in ER-negative breast cancers. Further investigation is essential to determine whether AIB1 might be effective therapeutic targets for ER-negative breast cancers, since positive IHC staining for AIB1 was observed in 53.8% of ER-negative breast cancers.

## Abbreviations

AIB1: amplified in breast cancer 1; ER: estrogen receptor; PR: progesterone receptor; SISH: silver *in situ *hybridization; AR: androgen receptor; ISH: *in situ *hybridization; IHC: immunohistochemistry; DAB: 3-3'-diaminobenzidine.

## Competing interests

The authors declare that they have no competing interests.

## Authors' contributions

AL and CSK conceived the study. KL performed the staining. BJS collected the cases and clinical information. KL and AL interpreted the staining results and performed the statistical analysis. KL performed the literature review and wrote the manuscript. AL and CSK supervised the experiments and manuscript writing. All authors read and approved the final manuscript.
